# Do you remember? Within-generation and transgenerational heat stress memory of recurring marine heatwaves in threespine stickleback

**DOI:** 10.1098/rspb.2024.2913

**Published:** 2025-02-05

**Authors:** Helen C. Spence-Jones, Cassandra Scheibl, Carla M. Pein, Monica Ionita, Lisa N. S. Shama

**Affiliations:** ^1^ Coastal Ecology Section, Alfred Wegener Institute Helmholtz Centre for Polar and Marine Research, Wadden Sea Station Sylt, Hafenstrasse 43, List 25992, Germany; ^2^ Department of Animal Physiology, Universität Bayreuth, Universitätstrasse 30, Bayreuth 95447, Germany; ^3^ Institute of Marine Ecosystem and Fishery Science, Universität Hamburg, Olbersweg 24, Hamburg 22767, Germany; ^4^ Ecological Chemistry Section, Alfred Wegener Institute Helmholtz Centre for Polar and Marine Research, am Handelshafen 12, Bremerhaven 27570, Germany; ^5^ Paleoclimate Dynamics Section, Alfred Wegener Institute Helmholtz Centre for Polar and Marine Research, am Handelshafen 12, Bremerhaven 27570, Germany

**Keywords:** marine heatwaves, heat stress memory, transgenerational plasticity, fitness-related traits, grandparent effects, stickleback (*Gasterosteus aculeatus*)

## Abstract

Marine heatwaves can have major and lasting effects on organism physiology and species persistence. Such temperature extremes are increasing in frequency, with consecutive heatwave events already occurring within the lifetime of many organisms. Heat stress memory (thermal priming) by individuals is a potential within-generation response to cope with recurring marine heatwaves. However, whether this form of biological memory can be inherited across generations is not well known. We used a three-generation experiment to investigate individual and transgenerational effects of single and recurring marine heatwaves on fitness-related traits using stickleback (*Gasterosteus aculeatus*) as a model species. We exposed adults (both sexes) to heatwaves and assessed female reproductive output in both the parent and offspring generation, and offspring (both sexes) survival, growth and behaviour to establish a holistic picture of potential heatwave effects on ectothermic fish. Exposure to single, extreme heatwaves lowered reproductive output, decreased offspring exploratory behaviour, impeded capacity to respond to further thermal stress and reduced long-term survival. However, prior experience of heatwaves (heat stress memory) mitigated some of these effects at both an individual (growth) and transgenerational (fecundity) level, indicating that species experiencing increasing heatwave frequency as part of ongoing climate change may cope better than previously thought.

## 1. Introduction

Marine heatwaves are increasing in intensity, frequency and duration under climate change [1,2], and even short periods of unusually high temperatures may have major effects on organisms [3,4]. Marine organism response to heatwaves may vary based on many factors, both extrinsic (such as the intensity, duration, timing and frequency of the heatwaves) and intrinsic (such as species, age, genetics, condition and prior thermal history) [5–7]. In extreme cases, heatwaves can result in mass mortality events and major ecosystem shifts that last long beyond the event itself [3,4,8] (but see [9]). While there are increasing field and laboratory studies examining these factors, relatively few investigate effects of recurring heatwave events (but see [10,11]) or long-term impacts on organisms that manifest well after heatwave exposure [12]. It is vital to understand the dynamics of individuals and species in response to such transient extremes, particularly in the context of recurring events, as these can have fitness consequences both within and across generations.

The majority of marine organisms are ectothermic and do not fully regulate their internal temperature, meaning that they must use behavioural strategies to avoid suboptimal temperatures [13,14], physiological adjustments to alter patterns and rates of heat transfer [15], or acclimation mechanisms to maintain physiological and metabolic functioning despite suboptimal temperatures (e.g. [16,17]). Even when effective, these mechanisms may incur time, risk, or energetic costs [18]. Where organisms cannot fully acclimate, acute thermal stress may result in deleterious effects on physiology, metabolism and immune function [7,19–21], reduction in reproductive output [7], and in extreme cases, death [5]. Ectotherms may be particularly vulnerable to mortality resulting from heatwaves [22], but even non-lethal effects can have significant impacts [10,11]. While some organisms show complete recovery following return to baseline temperatures [23,24], others may show long-lasting consequences [25].

Such lasting alterations to an organism’s physiology as a result of a heatwave may generate a form of biological memory that alters the response to subsequent temperature change [26–28] or other stressors [19]. Heat stress memory (thermal priming) occurs when exposure to sub-lethal high temperatures can expand an individual’s thermal tolerance range and improve fitness at high temperatures [29–31], for example by upregulation of protective heat-shock proteins [32]. This may result in better-than-expected performance in heatwaves for organisms that have been previously exposed to periods of unusually high temperature. However, it is also possible that previous exposure to stressful conditions depletes an organism’s resources and resilience, leaving them more vulnerable to additional stress [27]. Moreover, acclimation responses that maintain fitness during a heatwave event may nevertheless incur ‘ecological debts’, with costs only manifesting in the long term [33].

Another form of biological memory that may affect organism response to stress is transgenerational plasticity. Parental exposure to stress can lead to the inheritance of non-genetic factors that influence offspring phenotype [34,35], allowing information transfer about recent ancestral environmental conditions to be passed from parent to offspring. This may be adaptive or maladaptive [36–38], depending on the nature of factors transferred and the accuracy of prediction between parent and offspring environment. While these dynamics are beginning to be better understood [39–43], it remains unclear whether and how transgenerational plasticity may occur in response to transient extreme events, rather than shifts in the means of environmental variables. For example, in sea urchins, beneficial effects of heatwave-exposed parents on offspring thermal tolerance were restricted to early life stages and became deleterious if juveniles were exposed to further thermal stress in the post-settlement stage [44]. Similarly, exposure to high levels of temperature variation was found to be deleterious to fish growth, and mitigating effects of having parents that had experienced temperature variation were restricted to early life [45].

In this study, we assessed individual and transgenerational effects of single and recurring marine heatwaves on a model fish species, the threespine stickleback (*Gasterosteus aculeatus*). Ecologically relevant heatwave scenarios were modelled based on Hobday’s definition of 5 days with temperatures above the 90th percentile [46], and using time series data [47] of locally occurring heatwaves. We investigated the potential for heat stress memory on both an individual and transgenerational level by using two heatwave treatments: a single, extreme heatwave (+4.1°C for 11 days), and a double-heatwave (DHW) treatment where the extreme heatwave was preceded by a moderate heatwave (+2.6°C for 5 days). Maximum heatwave temperatures in the experiment (23°C) aligned with maximum temperatures experienced locally by this population [47]. We exposed adult males and females to the heatwave scenarios and took a holistic approach by measuring effects of heatwaves on female reproductive output (clutch size and egg size) of the F0 parent generation, as well as growth, survival and behaviour of F1 offspring (both sexes). We further assessed reproductive output of F1 adult females to test for effects of (grand)parent exposure to heatwaves on the F2 generation. We predicted that heatwaves would negatively affect some (or all) of these aspects of individual fitness, but that heat stress memory or transgenerational effects may increase the resilience of individuals exposed to heatwaves directly or whose (grand)parents had been exposed to heatwaves, and therefore be able to mitigate some of these negative effects.

## 2. Material and methods

### (a) Modelling heatwave treatments

Three temperature treatments were used: control (natural temperature variation; C), single-heatwave (SHW) and DHW. The control treatment was designed to reflect actual temperatures experienced by this population and was modelled using sea surface temperature data from a long-term temperature times series [47] at the Sylt-Rømø Bight, Germany (55.05N, 8.41E) and local buoy data (COSYNA data portal; https://www.hereon.de/institutes/carbon_cycles/cosyna/data_management/index.php.en; see electronic supplementary material, Methods). Heatwave treatments were identical to the control other than the occurrence of one ‘extreme’ (SHW) or one ‘moderate’ followed by one ‘extreme’ heatwave (DHW). Heatwaves were modelled using actual heatwave occurrences in the Sylt-Rømø Bight (see electronic supplementary material, Methods) and using Hobday’s [46] definition of marine heatwaves as a period of five or more days with temperatures warmer than the 90th percentile. The ‘moderate’ heatwave lasted 6 days with 1 day lead-in and lead-out (1°C increase/decrease per day) and was on average 2.62 ± 0.40°C higher than control temperatures. The ‘extreme’ heatwave lasted 14 days with 2 days lead-in and lead-out (1.5°C increase/decrease per day) and was on average 4.10 ± 0.57°C higher than control temperatures (electronic supplementary material, Methods).

### (b) Stickleback acclimation and breeding experiments

Our experiment encompassed three generations of stickleback: wild-caught F0 adults, their laboratory-bred F1 offspring which were reared to adulthood, and their F2 offspring (electronic supplementary material, figure S2). F0 wild adult fish were caught in the Sylt-Rømø Bight using trawling between 3 February and 10 March 2022 and immediately transferred to the laboratory [48]. Adults were randomly assigned to 25L aquaria on a flow-through seawater system (filtered seawater; pH = 7.85 ± 0.02, O_2_ = 99.2–100% saturation, salinity = 28.8 ± 0.5 ppt, flow rate = 0.15–0.4 l min^−1^) and a 12:12 L:D cycle with no more than 20 individuals per aquaria (*n* = 285 F0 adults split among 15 aquaria). Temperatures in the flow-through system were regulated using electronic thermostats connected to header tanks containing heaters (T-computer and 2 TH-500 heaters, Aqua-Medic, Bissendorf, Germany). Temperatures were initially maintained at 7°C. Adults were fed frozen bloodworms daily and held in laboratory conditions for a minimum of 2 days before temperature acclimation began, with temperature increases of 1°C per day until the experimental treatments (control) began 10 days later (as in [45]). No fish died during this temperature acclimation phase, despite the relatively fast change from late winter to early summer temperature conditions (see electronic supplementary material methods for the annual temperature profile), suggesting that the acclimation phase was not stressful. Throughout the experiment, water temperatures in the control and heatwave treatments (*n* = 5 replicate aquaria per treatment) were changed daily. Heatwaves occurred after 14 days (moderate) and 34 days (extreme) post-treatment onset. All adults (both sexes) were exposed to heatwave (or control) treatments. After 72 days, the light cycle was switched to 14:10 L:D to encourage adults to enter breeding conditions (distinctive colouration for males, gravid state with ‘pebbled’ appearance for females). Breeding began 2 weeks after the light cycle was switched. Standard length of breeding adults was measured using photography against a calibrated background (Canon EOS 650D).

F1 offspring families were generated using *in vitro* fertilization from F0 adults using embryo medium in petri dishes (see [49] for details). Egg clutches were fertilized by males within the same treatment, and all families were produced within an 11 days period (control *n* = 10 families, SHW *n* = 11, DHW *n* = 12). Fertilized clutches were monitored and maintained using daily water changes, with spoiled eggs recorded and removed, until hatching. Upon hatching, each clutch (family) was evenly split among the three treatments (electronic supplementary material, figure S2) with 10 individuals per family/treatment combination transferred to 1L aerated beakers of microfiltered seawater. Up to 10 additional individuals per family/treatment were transferred to secondary beakers for additional analyses. Offspring were fed *Artemia nauplii ad libitum*, and water in the beakers was exchanged weekly. After 30 days, offspring in beakers were transferred to 2L aquaria supplied by the flow-through seawater system. Offspring in secondary beakers were also transferred to secondary aquaria for future sampling. Within the relevant offspring heatwave treatments, heatwave events began at 34–45 days (moderate heatwave) and at 64–75 days (extreme heatwave) post-hatch. Feeding was changed from *Artemia nauplii* to frozen bloodworms when offspring reached 90 days post-hatch. F1 fish were transferred to 25L flow-through aquaria after 150 days post-hatch and maintained at control conditions (natural temperature variation) to monitor long-term survival. Each 25L aquaria contained all remaining fish within a family/treatment group (i.e. including those from secondary aquaria), with an average of 10.8 ± 3.9 individuals per 25L aquaria (range of 3–14).

F1 adult reproductive output was estimated using breeding individuals in their second year of life (between 19 April and 28 May 2024, mean age 685 ± 12 days), using the same methods as for F0 fish. All F1 gravid females were stripped of eggs, and clutch size and egg size were measured on all clutches (F0:F1 C:C *n* = 5, C:DHW *n* = 12, C:SHW *n* = 3, SHW:C *n* = 3, SHW:SHW *n* = 11, SHW:DHW *n* = 9, DHW:C *n* = 8, and DHW:SHW *n* = 9). Note: no F2 DHW:DHW clutches were produced. Due to logistic constraints, a full-factorial F2 breeding design (using all 9 F0:F1 treatment combinations) was not possible. Instead, only clutches from females in the F1 control treatment were fertilized using unrelated males within the same F0:F1 treatment combination (i.e. no between treatment crosses were made). Using only control group F1 parents allowed us to test for possible grandparent (F0) effects on F2 offspring traits. Fertilization success, hatching success and hatchling size were assessed using only these clutches (electronic supplementary material, figure S2).

### (c) Metrics assessed

All statistical analyses were performed in the R v. 4.3.2 [50] statistical environment using the packages ‘dplyr’, ‘forcats’, ‘PMCMRplus’, ‘nlme’, ‘lme4’, ‘pylr’, ‘MASS’, ‘lawstat’, ‘heatwaveR’, ‘ggsurvfit’ and ‘survival’, and graphs were produced using the package ‘ggplot2’. We modelled most metrics using general linear models or linear mixed-effect models with Gaussian error distributions and an identity link. If assumptions of general linear models were not met (e.g. data not normally distributed), we used Kruskal–Wallis (KW) tests.

#### (i) F0 and F1 reproductive output: clutch size and egg size

Egg clutches were photographed under a microscope (Stemi 503, 6.3× magnification) against a calibrated background. Fecundity (clutch size) was assessed as the total number of eggs within a clutch. Clutch size was compared among treatments using a general linear model with maternal standard length and parent treatment as fixed factors. Egg size was estimated upon fertilization by measuring the maximum diameter of all eggs in a clutch (using ImageJ [51]). Egg diameter was compared across treatments using a linear mixed-effect model with parent treatment, clutch size and the interaction between the two as fixed factors, and clutch ID (*n* = 33) as a random factor.

Fertilization success was estimated as the percentage of a clutch which was developing normally 1 day post-fertilization. Fertilization success was compared among treatments using a KW test as residuals were not normally distributed (Shapiro–Wilk *W* = 0.672, *p* < 0.001). One F0 clutch had a fertilization success below 40% and was removed from all analyses. Therefore, 32 F0 clutches were analysed (control *n* = 10, SHW *n* = 11, DHW *n* = 11). Hatching success was calculated as the percentage of fertilized eggs within a clutch that successfully hatched into fry that survived at least 1 day post-hatch. Hatching success was compared among parental treatments using a KW test (Shapiro–Wilk *W* = 0.654, *p* < 0.001).

The rate of females going into breeding condition was assessed for F1 adults as the percentage of females in each aquarium that went into breeding condition (within the breeding period) using a general linear model with parent treatment, offspring treatment and their interaction as factors. Sex ratio at the point of F1 breeding was also compared among treatments by calculating the percentage of females per aquarium and comparing across treatments using a general linear model with parent treatment, offspring treatment and their interaction as factors. In both cases, aquaria with only one fish remaining were excluded from analyses.

In the F1 adult generation, clutch size and egg size were modelled using the same approach as F0 adults, but with grandparent (F0) treatment, parent (F1) treatment and their interaction as fixed factors, and clutch ID (*n* = 60) as a random factor in the egg size model. F2 fertilization success, hatching success and hatchling length were only compared among clutches with control parents to assess effects of grandparent (F0) treatment. Fertilization and hatching success were modelled using KW tests (as for F0 adults), and hatchling length was modelled using a linear mixed-effect model with F0 treatment and egg diameter as fixed effects, and clutch ID (*n* = 15) as a random effect.

#### (ii) F1 survival and growth

Short-term survival was assessed using offspring survival up to 90 days (measured at 30, 60 and 90 days) with a Cox proportional hazard model containing parent treatment, offspring treatment and their interaction as factors. Long-term survival was assessed following transfer of offspring into 25L aquaria (after the initial 90 days experiment) using a Cox proportional hazard model containing parent treatment, offspring treatment and their interaction as factors. Long-term survival was assessed at 153, 409, 611 and 717 days post-hatch.

F1 offspring length was measured at 1, 30, 60, and 90 days post-hatch using the 10 individuals in each primary beaker/aquarium to control for density effects. Hatchlings were measured 1 day post-hatch by photographing petri dishes containing hatchlings against a calibrated background (Canon EOS 650D). Older offspring were measured by photographing individuals on a calibrated background under a microscope (Stemi 503, 6.3× magnification). ImageJ [51] was used to measure the standard length of all photographed fish. Hatchling length was compared among treatments using a linear mixed-effect model with parent treatment and clutch-average egg diameter as fixed effects, and clutch ID (*n* = 31) as a random effect. In all other growth analyses, individual length was compared among treatment combinations using linear mixed-effects models with parent treatment, offspring treatment, and their interaction as fixed effects, and clutch ID (*n* = 31) as a random effect.

#### (iii) F1 behaviour

Behavioural metrics were assessed on a subset of 30 individuals from four F0 control split-clutch families (F1 offspring at control *n* = 12, F1 offspring at SHW *n* = 7, F1 offspring at DHW *n* = 11) at between 629 and 645 (mean 636) days post-hatch. Activity rate and exploratory behaviour were compared among individuals using open-field tests. Tests were conducted on individual fish in a circular arena (78 cm diameter, water depth 8 cm) lined with a white cotton cloth to generate sloped sides and weighted with a 0.5 cm layer of white sand. Experimental arenas were enclosed within dark areas and lit from above using LED strip lights. Water temperature was maintained between 16 and 17°C during tests. Fish behaviour throughout both experiments was recorded with video cameras mounted above the arena (ELP 5.0 Megapixel USB Camera). A 10 × 10 grid was overlaid onto the arena and the grid-square of the fish recorded in each frame. Fish were placed individually in a starting chamber (11 cm diameter transparent perforated plastic). After a 1 min acclimation period, the fish was released from the starting chamber and allowed to explore the arena for 15 min. Three arenas were used simultaneously to assess replicate fish from the same aquaria.

Videos were analysed using automated tracking software (eZTrack [52]) to extract positional data of the fish at a resolution of four frames per second. Anonymized tracked videos were then watched by a single human observer to verify fidelity of the tracking. Any time periods where the trace was lost were manually removed from the data. Two metrics of behaviour were used: activity rate (average speed over the entire trial period, calculated as cm moved per second) and exploration (percent of arena explored). To assess exploration, the cumulative percentage of grid-squares that had been entered by the fish was calculated over time for each fish. Activity rate was compared among offspring treatments using a KW test. Exploratory behaviour was compared among offspring treatments using a linear mixed-effect model, with offspring treatment, the third-order polynomial of time, and their interaction as fixed effects, and clutch ID (*n* = 4) of the fish as a random factor.

## 3. Results

### (a) Reproductive output (F0 generation)

**Figure 1.**
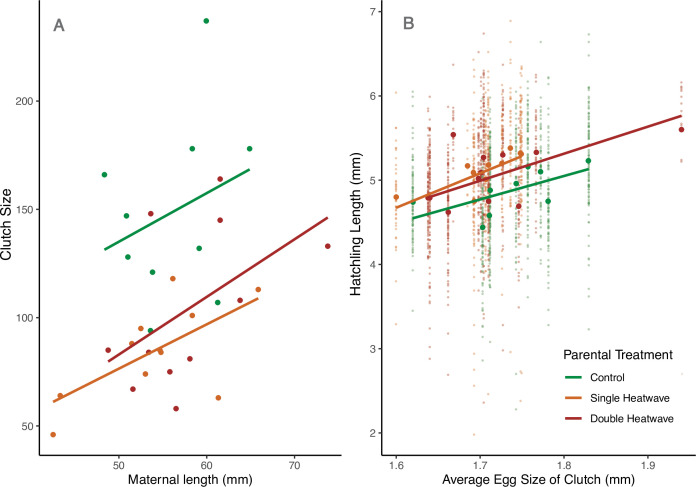
Parent (F0) stickleback (*Gasterosteus aculeatus*) (*A*) clutch size as a function of maternal size (standard length) and (*B*) hatchling length as a function of egg size in the three parent heatwave treatments.

Fecundity (clutch size) was reduced for mothers exposed to heatwaves during late gametogenesis (in the 2 months prior to breeding). On average, heatwave-exposed mothers (both SHW and DHW) laid fewer eggs than mothers of equivalent size in the control treatment (control mean clutch size = 148.8 ± 42.2 eggs, SHW = 84.6 ± 21.9 eggs, DHW = 104.4 ± 37.0 eggs; table 1; figure 1). Clutch sizes from mothers in SHW or DHW were not significantly different to each other (*t* = −0.739, *p* = 0.466), implying no additive effect of days-of-heatwave exposure on fecundity. Size of breeding adults was not significantly different among treatment groups for males (KW Χ^2^_(2)_ = 3.105, *p* = 0.212) or females (KW Χ^2^_(2)_ = 0.773, *p* = 0.679). Variation in size was also not significantly different for males (Brown–Forsythe Levene’s (BFL) test statistic = 0.398, *p* = 0.675) or females (BFL = 0.415, *p* = 0.662).

**Table 1.** Linear models of stickleback (*Gasterosteus aculeatus*) F0 clutch size, egg diameter and hatchling standard length as a function of parent (F0) heatwave treatment (SHW = single heatwave, DHW = double heatwave) and covariates. Clutch ID was included as a random factor in models of egg diameter and hatchling length.

clutch size (no. eggs)			egg diameter (mm)			hatchling length (mm)		
	value ± s.e. (mm)	*p*‐value		value ± s.e. (mm)	*p*‐value		value ± s.e. (mm)	*p*‐value
F0: control (intercept)	17.9 ± 51.9	0.732	F0: control (intercept)	1.7937 ± 0.0458	<0.001	F0: control (intercept)	0.05 ± 1.19	0.969
maternal length (mm)	2.3 ± 0.9	0.016	clutch size (no. eggs)	−0.0004 ± 0.0003	0.207	egg diameter (mm)	2.78 ± 0.69	<0.001
F0: SHW	−59.2 ± 13.9	<0.001	F0: SHW	−0.0721 ± 0.0294	0.020	F0: SHW	0.33 ± 0.10	0.004
F0: DHW	−48.9 ± 13.9	0.002	F0: DHW	−0.0571 ± 0.0276	0.047	F0: DHW	0.24 ± 0.10	0.022
clutch ID (random effect) s.d., residual s.d.			0.059, 0.062			0.211, 0.552		

Stickleback mothers in heatwave treatments also laid significantly smaller eggs than control mothers, and this was independent of clutch size. Eggs from mothers in heatwave treatments were on average 0.052 mm smaller than those from control mothers (table 1). Intra-clutch egg size variation was not significantly different among parent treatment groups (KW Χ^2^_(2)_ = 2.847, *p* = 0.241), nor was inter-clutch egg size variation (BFL = 0.807, *p* = 0.456).

Fertilization success was generally high (mean 92.3 ± 13.3%). Fertilization and hatching success showed no significant differences among treatments (fertilization: KW Χ^2^_(2)_ = 2.128, *p* = 0.345; hatching: KW Χ^2^_(2)_ = 5.752, *p* = 0.056). However, there was a non-significant trend (Dunn post-hoc *p* = 0.089) for clutches of single heatwave parents to show lower hatching success than those of control parents (control = 90.4 ± 16.9%; SHW = 75.6 ± 30.0%; DHW = 87.2 ± 14.1%).

Egg size was significantly correlated with hatchling size, with larger eggs resulting in larger hatchlings across all treatments. However, despite producing smaller eggs on average, heatwave parents produced significantly larger hatchlings than expected (table 1; figure 1), resulting in significantly larger hatchlings in heatwave treatments overall, with the largest hatchlings for SHW parents (KW Χ^2^_(2)_ = 69.232, *p* < 0.001; Dunn post-hoc *p* < 0.001 for all pairings: control 4.87 ± 0.60 mm, SHW 5.12 ± 0.62 mm, DHW 4.97 ± 0.60 mm). This result was robust to the removal of a DHW clutch with unusually large egg size (KW Χ^2^_(2)_ = 70.187, *p* < 0.001; Dunn post-hoc control:DHW *p* = 0.001, *p* < 0.001 for all other pairings).

### (b) Offspring survival

Offspring short-term survival (in the first 90 days post-hatch) was universally high, with an average of 94.4% survival across all family/treatment combinations. Short-term survival was not significantly affected by parent heatwave treatment, offspring heatwave treatment, or the interaction between the two (Cox likelihood ratio test_(8)_=6.95, *p* = 0.542). However, long-term survival (between 91 and 720 days post-hatch) was significantly affected by both parent and offspring treatments (Cox likelihood ratio test_(8)_ = 34.98, *p* < 0.001; table 2; electronic supplementary material, figure S3). Offspring of both SHW and DHW parents were significantly more likely to die earlier than those with control parents, and offspring individuals exposed directly to a SHW had significantly lower survival rates (table 2). Long-term survival was also significantly influenced by the interaction between parent and offspring generation heatwave exposure, in that SHW individuals who had a heatwave parent, particularly those with a DHW parent, had higher survival than controls (table 2). Mean long-term survivorship in the nine parent:offspring treatment combinations was: C:C 0.76 ± 0.15, C:SHW 0.56 ± 0.31, C:DHW 0.72 ± 0.30, SHW:C 0.53 ± 0.24, SHW:SHW 0.59 ± 0.42, SHW:DHW 0.52 ± 0.29, and DHW:C 0.49 ± 0.26, DHW:SHW 0.66 ± 0.23, DHW:DHW 0.46 ± 0.28 (electronic supplementary material, figure S3).

**Table 2.** Cox regression analysis of stickleback long-term survival probability as a function of parent (F0) and offspring (F1) heatwave treatment (SHW = single heatwave, DHW = double heatwave), and their interaction. Coefficients refer to probability of death, with negative coefficients indicating higher survival probability.

	coefficient ± s.e.	*p*‐value
F0:SHW	0.85 ± 0.27	0.001
F0:DHW	0.99 ± 0.26	<0.001
F1:SHW	1.02 ± 0.27	<0.001
F1:DHW	0.39 ± 0.29	0.174
F0:SHW—F1:SHW	−0.93 ± 0.35	0.009
F0:SHW—F1:DHW	−0.25 ± 0.36	0.480
F0:DHW—F1:SHW	−1.26 ± 0.34	<0.001
F0:DHW—F1:DHW	−0.24 ± 0.35	0.494

### (c) Offspring growth

**Figure 2.**
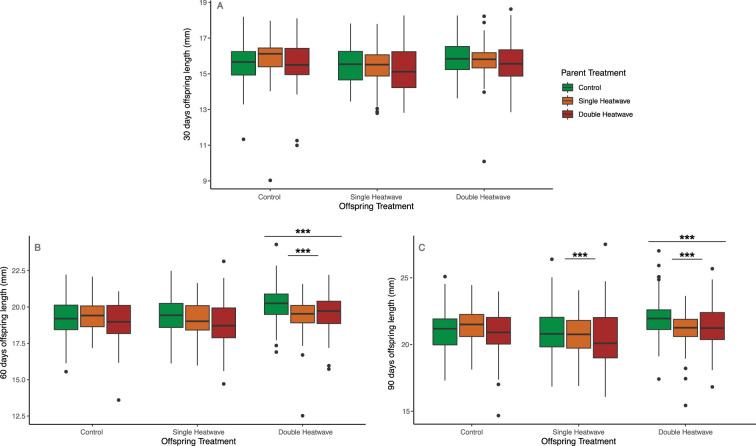
Offspring (F1) stickleback (*Gasterosteus aculeatus*) standard length (mm) at (*A*) 30 days, (*B*) 60 days and (*C*) 90 days post-hatch in the nine parent:offspring (F0:F1) heatwave treatment combinations.

At 30 days post-hatch, all F1 offspring (*n* = 799) had experienced the same temperature regime, and there were no significant differences in length among treatment groups, including parent treatment groups (figure 2; table 3). At 60 days post-hatch (*n* = 795), offspring in the DHW treatment were the only ones to have experienced different temperatures (a moderate heatwave), and were significantly larger than either SHW or control fish (1.01 mm larger than control). There was a significant parent x offspring heatwave interaction, with offspring of SHW parents being significantly smaller than expected in the DHW treatment (−1.00 mm; table 3). At 90 days post-hatch (*n* = 794), offspring in the double heatwave treatment maintained their significantly larger size (0.95 mm larger than control). Offspring of SHW parents were significantly smaller than expected in both SHW (−0.65 mm) and DHW treatments (−1.27 mm; figure 2; table 3).

**Table 3.** Linear mixed-effect models of stickleback (*Gasterosteus aculeatus*) offspring standard length at 30, 60 and 90 days post-hatch as a function of parent (F0) and offspring (F1) heatwave treatments (SHW = single heatwave, DHW = double heatwave) and their interaction.

	30 day length		60 day length		90 day length	
	value ± s.e. (mm)	*p*‐value	value ± s.e. (mm)	*p*‐value	value ± s.e. (mm)	*p*‐value
F0/F1: control (intercept)	15.62 ± 0.21	<0.001	19.19 ± 0.21	<0.001	21.00 ± 0.27	<0.001
F0:SHW	0.26 ± 0.31	0.416	0.18 ± 0.32	0.572	0.47 ± 0.41	0.267
F0:DHW	−0.02 ± 0.29	0.950	−0.19 ± 0.30	0.536	−0.12 ± 0.39	0.764
F1:SHW	−0.10 ± 0.14	0.493	0.20 ± 0.18	0.255	−0.03 ± 0.21	0.884
F1:DHW	0.21 ± 0.14	0.142	1.01 ± 0.17	<0.001	0.95 ± 0.21	<0.001
F0:SHW— F1:SHW	−0.31 ± 0.21	0.135	−0.39 ± 0.26	0.143	−0.65 ± 0.31	0.037
F0:SHW— F1:DHW	−0.37 ± 0.21	0.076	−1.00 ± 0.26	<0.001	−1.27 ± 0.31	<0.001
F0:DHW— F1:SHW	−0.23 ± 0.20	0.255	−0.35 ± 0.25	0.164	−0.38 ± 0.30	0.193
F0:DHW— F1:DHW	−0.20 ± 0.20	0.324	−0.44 ± 0.25	0.076	−0.46 ± 0.30	0.117

### (d) Offspring behaviour

#### (i) Activity rate

There was no significant difference in activity rate (average speed) among offspring treatment groups (KW Χ^2^_(2)_ = 0.293, *p* = 0.864).

#### (ii) Exploration

**Figure 3.**
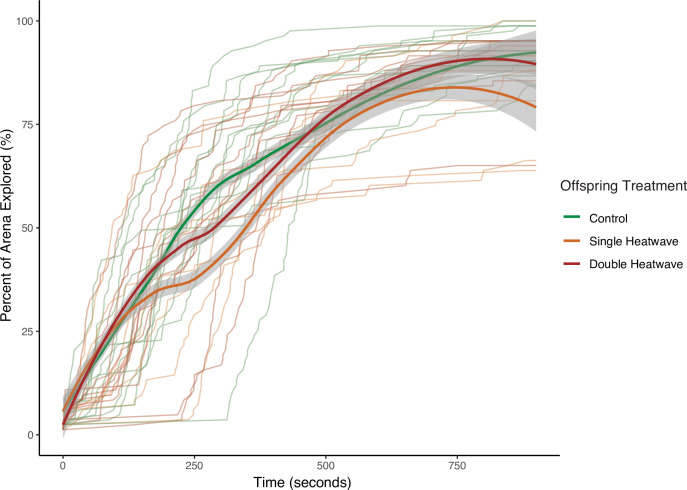
Exploratory behaviour (percent of the experimental arena explored) of F1 stickleback (*Gasterosteus aculeatus*) over the course of the observation period. Faint lines represent individual tracks, while bold lines represent group averages with 95% CIs.

There were significant differences in exploratory behaviour among treatments, with both DHW and SHW offspring fish exploring significantly less (DHW: −1.3 grid squares, *p* = 0.037; SHW: −5.4 grid squares, *p* < 0.001) than control fish overall (electronic supplementary material, table S1). Heatwave fish also showed a different pattern of exploration to control fish (figure 3), with SHW fish showing slightly higher exploratory rates within the first 100 s (2.6 grid squares, *p* = 0.006), and DHW fish showing no differences in exploration from control fish within this time (*p* = 0.758; electronic supplementary material, table S1).

### (e) F1 generation reproductive output

There were no significant differences in F1 adult sex ratio (*F*_(8,62)_=1.021, *p* = 0.430) or rate of F1 females going into breeding condition among most treatment groups, with the exception of SHW individuals with SHW (F0) parents, which went into breeding condition more frequently than expected (estimate = 60.089 ± 28.742; *t*_(1)_=2.091; *p* = 0.041). In some treatment combinations, there were relatively few clutches produced (*n* = 3 for C:SHW and SHW:C; other treatment combinations *n* = 5 to *n* = 12), and no successful clutches were produced in the DHW:DHW treatment.

**Figure 4.**
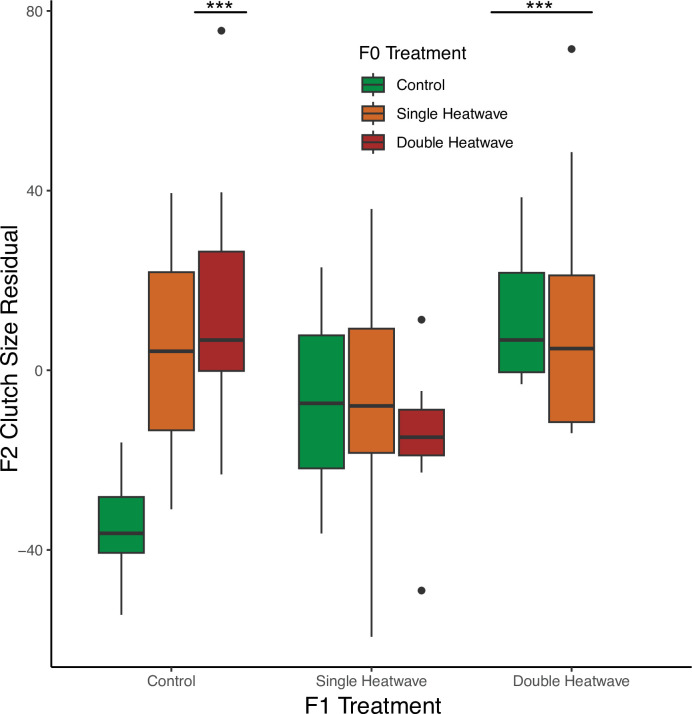
Fecundity of F1 adult female stickleback in terms of F2 clutch size as a function of F0 grandparent and F1 parent heatwave treatment (residuals standardized by maternal length).

Fecundity (clutch size) was significantly influenced by F1 parent and F0 grandparent exposure to double heatwaves, in both cases resulting in significantly larger clutches than mothers (or grandparents) that experienced SHW or control treatments (table 4, figure 4). However, this effect was negated for individuals with DHW grandparents if they were raised in the SHW treatment (F0 × F1 interaction; table 4). Egg size was significantly impacted by parental heatwave treatment but not grandparent heatwave treatment (table 4). F1 females with both SHW and DHW parents produced significantly smaller eggs than those with control parents. As found for the F0 clutches, egg size was not significantly influenced by clutch size (table 4).

**Table 4.** Linear models of stickleback (*Gasterosteus aculeatus*) F1 adult fecundity (clutch size) and egg diameter, and F2 hatchling length as a function of maternal length and individual/parent (F1) and grandparent (F0) heatwave treatments (SHW = single heatwave, DHW = double heatwave).

clutch size (no. eggs)			egg diameter (mm)			hatchling length (mm)		
	value ± s.e. (mm)	*p*		value ± s.e. (mm)	*p*		value ± s.e. (mm)	*p*
F0:control (intercept)	−60.4 ± 51.4	0.245	F0:control (intercept)	1.8526 ± 0.0325	<0.001	F0:control (intercept)	1.48 ± 1.99	0.458
maternal length (mm)	2.0 ± 0.9	0.033	clutch size (no. eggs)	−0.0003 ± 0.0003	0.372	egg diameter (mm)	1.91 ± 1.08	0.105
F0:SHW	40.0 ± 20.9	0.061	F0:SHW	−0.0430 ± 0.0469	0.363	F0:SHW	0.14 ± 0.25	0.571
F0:DHW	49.4 ± 14.5	0.001	F0:DHW	−0.0775 ± 0.0400	0.058	F0:DHW	0.23 ± 0.21	0.295
F1:SHW	28.2 ± 18.2	0.127	F1:SHW	−0.0937 ± 0.0464	0.049	all hatchlings were in F1: control		
F1:DHW	45.9 ± 13.3	0.001	F1:DHW	−0.1311 ± 0.0372	<0.001			
F0:SHW—F1:SHW	−37.5 ± 26.7	0.166	F0:SHW—F1:SHW	0.0411 ± 0.0627	0.515			
F0:SHW—F1:DHW	−36.4 ± 23.8	0.133	F0:SHW—F1:DHW	0.0683 ± 0.0549	0.220			
F0:DHW—F1:SHW	−57.1 ± 22.2	0.013	F0:DHW— F1:SHW	0.0522 ± 0.0586	0.377			
F0:DHW—F1:DHW	NA	NA	F0:DHW— F1:DHW	NA	NA			
clutch ID (random effect) s.d., residual s.d.			0.004, 0.007			0.279, 0.383		

While fertilization success did not significantly differ among F1 parents (average fertilization success 97.3±5.5%; KW Χ^2^_(1)_ = 0.769, *p* = 0.381), clutches from SHW parents showed significantly lower hatching success than those from control parents (control parent: 80.7 ± 22.9%, SHW parent: 59.6±26.8%; KW Χ^2^_(1)_ = 5.878, *p* = 0.015). Grandparent heatwave treatment did not have a significant influence on either fertilization success (KW Χ2_(2)_ = 1.936, *p* = 0.380) or hatching success (KW Χ2_(2)_ = 1.682, *p* = 0.431). F2 hatchling size was not significantly different among grandparent treatments (table 4) and was positively correlated with (clutch average) egg size.

## 4. Discussion

Our results present a holistic picture of stickleback response to heatwaves (electronic supplementary material, figure S4), showing that while heatwave events are stressful for individuals, heat stress memory (thermal priming) may mitigate this at both an individual and transgenerational level. We show that single, extreme heatwaves can lower reproductive output, decrease exploratory behaviour and impede offspring capacity to respond to further thermal stress, as well as reduce lifespan in the long term. However, prior experience of moderate heatwaves both within and across generations can mitigate some of the negative effects of extreme heatwave events.

Marine heatwaves have led to mass mortalities of fish populations around the globe [4]. In this experiment, although stickleback short-term survival was not immediately impacted by heatwaves, long-term survival was affected by both direct and parental experience of heatwaves. Individuals experiencing a single heatwave died earlier than those experiencing either DHWs or no heatwave. However, this effect was reduced for SHW individuals whose parents had experienced either SHW or DHW treatments, with higher survival than fish with control parents, suggesting potential transgenerational benefits of parental heatwave exposure on offspring long-term survival. For control treatment offspring, however, having heatwave parents significantly decreased the probability of long-term survival, suggesting that mismatch and a lack of predictability between parent and offspring environmental cues [53] associated with heatwaves may have contributed to negative transgenerational effects on long-term survival for those offspring. Although mortality resulting directly from or immediately following exposure to extreme temperatures is commonly reported (e.g. [25]), there are fewer studies examining long-term changes to survivorship resulting from transient exposure to heat stress. Data from wild Indo-Pacific bottlenose dolphins (*Tursiops aduncus*) suggests that survival may be impacted for at least seven years post-heatwave [54]. In contrast, no evidence for negative impacts of a thermal challenge on juvenile fish for either short- or long-term survival was found in wild brown trout (*Salmo trutta*) [55].

Heatwaves can have major consequences for all aspects of reproduction, including gamete development, fecundity, egg size, fertilization success and offspring growth [56]. We found that female reproductive output (clutch size and egg size) was negatively affected by heatwaves, particularly in the SHW treatment. Stickleback mothers who had experienced (single or double) heatwaves during gametogenesis put significantly less investment into their clutches, with both smaller clutches and smaller eggs. Clutch size is often traded off against egg size, with a common pattern of more, but smaller eggs produced under higher temperatures [57]. While larger egg size is often associated with higher quality offspring, smaller egg size may be adaptive at warmer temperatures due to reduced energetic demands [58]. Patterns in other species appear to be mixed, with coral reef damselfish (*Acanthochromis polacanthus*) producing smaller clutches but larger eggs following a heatwave [59], freshwater snails (*Lymnaea stagnalis*) producing smaller eggs [60] and purple sea urchins (*Stronglocentrotus purpuratus*) showing no change in egg size [61]. Interestingly, we found that despite smaller egg sizes, hatchlings with heatwave parents were significantly larger than those with control parents, implying that maternal provisioning or other non-genetic inheritance mechanisms (e.g. epigenetic modifications) may have led to alterations in resource allocation and/or gene expression [26,34]. For instance, differential nutrient conditions within eggs may have promoted compensatory growth during embryonic development, resulting in larger hatchlings from heatwave parents [62]. Similar responses have been found in the sea urchins *Helicidaris erythrogramma* [44] and *S. purpuratus* [61], both of which showed increased offspring size in heatwave-exposed mothers. In *S. purpuratus,* this was associated with increased protein concentrations in eggs, while egg size did not differ. In our study, beneficial effects on offspring size did not appear to carry over to early juvenile growth; however, as there were no significant differences in size at 30 days among offspring from different parental treatments, implying that beneficial transgenerational effects in response to temperature extremes may be short-term and transient (see also [45]).

Environmental conditions experienced by both parents can contribute to offspring phenotype. Examples of maternal environment effects on offspring traits are pervasive in the literature [43], but the role of paternal environment effects is gaining attention. For instance, changes to sperm quality in response to exposure to single heatwaves in male flour beetles (*Tribolium castaneum*) led to reduced fertility and sperm competitiveness, whereas female reproduction was unaffected. Moreover, exposure to recurring heatwaves resulted in male near-sterility [10]. Across generations, flour beetle fathers exposed to heatwaves had sons with decreased reproductive output and lifespan [10], and exposure during the pupal stage (last stage before adult emergence and mating) was a key exposure window [11]. In Atlantic salmon (*Salmo salar*), warmer temperatures during incubation benefited eggs but harmed sperm, resulting in lower hatching success and offspring fitness [63]. Likewise, sperm quality in purple sea urchins was reduced when males were exposed to heatwaves [64], affirming the thermal sensitivity of male gametes. In stickleback, paternal and grandpaternal exposure to higher (constant) temperatures during gametogenesis played a role in lower F2 offspring hatching success, indicating cumulative negative effects down the paternal line [65]. Here, although we did not address sperm performance explicitly, we found no apparent evidence of male sperm quality being affected by heatwaves, since we had consistent fertilization success across treatments. However, there is recent evidence that male stickleback breeding behaviour is negatively affected by prolonged heatwaves [6], and even short-term heatwaves experienced during breeding can have lasting consequences for parents and offspring [66].

For F1 adults, heatwave treatments also resulted in changes to female fecundity and egg size, despite the length of time that had elapsed between heatwave exposure and reproductive output assay (approx. 1.7 years). Egg sizes were smaller when mothers were exposed to heatwaves, but most interesting, the DHW treatment appeared to stimulate larger clutch sizes (independently of its effect on F1 offspring growth) for both F1 mothers directly experiencing the DHW treatment, and for those with parents (F0 grandparent) that experienced a DHW. This implies that early life exposure to heatwaves (occurring before reproductive maturity) as well as parental exposure to heatwaves may have long-term impacts on reproductive output [67], for both fecundity (here, larger clutch sizes in DHWs) and egg size (here, smaller eggs in heatwaves). These results are in contrast to a recent study of burying beetles (*Nicrophorus vespilloides*) where the effects of heatwave exposure differed among short windows (3 days stages) of the reproductive cycle, with smaller brood sizes only occurring if heatwaves were experienced during the mating stage [56]. Hence, fitness consequences of exposure to heatwaves are likely timing-dependent. Exposure before sexual maturity could primarily affect gametogenesis, and in turn, fecundity and fertilization success, whereas exposure during mating could affect sexual selection and mating interactions, and exposure after offspring hatch could affect offspring survival and growth [56]. Beneficial effects of heat stress memory to recurring heatwaves for individuals (within a generation) have recently been shown in a few studies (e.g. [25–28]), but consequences of transgenerational exposure to recurring marine heatwave events for fecundity of the next generation(s) is still not well known (but see [10]).

Whether recurring extreme events result in additive, synergistic or antagonistic organism responses is a key open question in marine ecosystems [68]. In corals, for example, responses to recurring heatwaves range from acclimation via environmental memory to sensitization [69]. In our experiment, offspring individuals who experienced a DHW reached the largest sizes overall. Higher temperatures experienced during the moderate heatwave (of the DHW treatment) facilitated growth in those fish, with differences apparent after that first heatwave and persisting following the second, more extreme heatwave. In contrast, individuals who were exposed to a single, extreme heatwave within their lifetime did not show a similar growth benefit. Moreover, having parents who experienced a single heatwave appeared to be deleterious when those offspring themselves experienced heatwave stress. Specifically, offspring of SHW parents were smaller than expected (after 90 days) in both SHW and DHW treatments, implying that negative effects of parental extreme heatwave stress manifest for offspring in stressful conditions, indicative of negative transgenerational effects of SHW events on offspring growth. Parents who experienced a DHW, on the other hand, did not negatively influence offspring growth. Those offspring did not differ in size from control-parent fish under either heatwave condition, possibly indicating some form of compensatory growth mechanism [62] and/or potential transgenerational heat stress memory benefit on offspring growth [26].

Changes to behaviour are often an individual´s first response to altered environmental conditions [66]. Despite no differences in activity rate, stickleback that had experienced heatwaves (particularly SHW fish) showed less exploratory behaviour than fish in the control treatment. Similarly, the Iberian barbel (*Luciobarbus bocagei*) showed lower activity and reduced boldness under heatwave conditions [70]. Other acute sources of stress have been associated with decreases in exploratory behaviour in fish, such as guppies (*Poecilia reticulata*) exposed to oil pollution [71], and zebrafish (*Danio rerio*) exposed to polycyclic aromatic hydrocarbons [72]. Still, chronic higher temperatures have been found to result in increased exploratory behaviour in largemouth bass (*Micropterus salmoides*) [73] and zebrafish [74]. In our study, it is unclear whether the observed reduction in exploratory behaviour observed many months after heatwave exposure implies cognitive impairment such as poor spatial memory [71] arising from transient experience of high temperatures, and/or whether it has fitness consequences in real-world conditions. More exploratory fish are better able to locate resources, but at a cost of energy, time, and exposure to predators [75,76]. As our fish were raised in controlled conditions with easily accessible food and no risk of predation, we could not assess this and saw no evidence of reduced growth for heatwave-exposed fish. Indeed, fish exposed to the DHW treatment showed increased growth relative to those that did not experience a heatwave. Regardless, the differences in exploratory behaviour observed between heatwave-exposed and non-heatwave-exposed fish months after heatwave exposure, implies that even short-term exposure to extreme temperature events may have long-lasting cognitive and/or behavioural effects.

## 5. Conclusion

Our results show a consistent pattern across organism responses that single, extreme heatwaves negatively impact individuals, and that parental experience of single, extreme heatwaves may reduce their offspring’s ability to both take advantage of good conditions and cope with stressful conditions. However, we found a heat stress memory (thermal priming) beneficial effect whereby the negative effects of an extreme heatwave could be mitigated by prior experience of a moderate heatwave. Specifically, offspring fish exposed to a double heatwave within their lifetime were larger than those exposed to a single heatwave. Also, negative parental effects caused by the SHW treatment did not apply to DHW parents. While offspring of SHW parents displayed reduced growth in both SHW and DHW conditions, offspring of DHW parents were comparable to those with control (no heatwave) parents. Furthermore, fecundity (clutch size) of F1 adults was higher if parents or grandparents experienced a double heatwave, implying that beneficial effects of heat stress memory may be transgenerational. Taken together, our results suggest that heat stress memory has the potential to increase thermal performance of organisms both within and across generations, and species experiencing an increased frequency of marine heatwaves as part of ongoing climate change may be better able to cope than previously thought.

## Data Availability

Data are available in the PANGAEA Data Publisher for Earth & Environmental Science [77]. Supplementary material is available online [78].
